# Investigations into the Performance of a Novel Pocket-Sized Near-Infrared Spectrometer for Cheese Analysis

**DOI:** 10.3390/molecules24030428

**Published:** 2019-01-24

**Authors:** Verena Wiedemair, Dominik Langore, Roman Garsleitner, Klaus Dillinger, Christian Huck

**Affiliations:** 1 CCB—Center for Chemistry and Biomedicine, Institute of Analytical Chemistry and Radiochemistry, University of Innsbruck, Innrain 80/82; 6020 Innsbruck, Austria; verena.wiedemair@uibk.ac.at (V.W.); dominik.langore@student.uibk.ac.at (D.L.); 2 Chemical devision, HBLFA für Landwirtschaft und Ernährung, Lebensmittel und Biotechnologie Tirol, Rotholz 50a, 6200 Strass im Zillertal, Austria; roman.garsleitner@hblfa-tirol.at (R.G.); klaus.dillinger@hblfa-tirol.at (K.D.)

**Keywords:** NIR, SCiO, pocket-sized spectrometer, cheese, fat, moisture, multivariate data analysis

## Abstract

The performance of a newly developed pocket-sized near-infrared (NIR) spectrometer was investigated by analysing 46 cheese samples for their water and fat content, and comparing results with a benchtop NIR device. Additionally, the automated data analysis of the pocket-sized spectrometer and its cloud-based data analysis software, designed for laypeople, was put to the test by comparing performances to a highly sophisticated multivariate data analysis software. All developed partial least squares regression (PLS-R) models yield a coefficient of determination (R^2^) of over 0.9, indicating high correlation between spectra and reference data for both spectrometers and all data analysis routes taken. In general, the analysis of grated cheese yields better results than whole pieces of cheese. Additionally, the ratios of performance to deviation (RPDs) and standard errors of prediction (SEPs) suggest that the performance of the pocket-sized spectrometer is comparable to the benchtop device. Small improvements are observable, when using sophisticated data analysis software, instead of automated tools.

## 1. Introduction

A wide variety of cheese and cheese products can be purchased in stores around the world. Cheese is an important source of nutrients, such as fat and protein [1], and is consumed worldwide with an annual production of approximately 23 million tonnes in 2014 [2]. Quality control is vital, to ensure food safety and to protect consumer interest. Hence, a wide variety of physico-chemical analyses were developed to determine pH, fat, nitrogen fractions, volatile fatty acids and others [3,4]. Traditional methods to determine these nutrients have some disadvantages. They are often time consuming, expensive and have a limited sample throughput [3,5]. Furthermore, trained personnel is needed to operate the machines and execute the analyses [6].

That is why, fast and non-destructive spectral methods were developed to measure main components, such as fat, protein and moisture, of cheese [3,7,8,9]. More recent approaches also focus on minor components, like vitamins, minerals and carotenoids [10,11]. But lately, near-infrared spectroscopy (NIRS) was also used to identify sensory properties, as well as the origin of cheese [12,13,14,15,16]. Cheese is not spatially homogeneous, which poses a challenge for spectral analysis [10].

NIRS is widely applied in food analyses [17,18,19,20], as well as in other fields including pharmaceutical sciences and petrochemistry [21,22,23,24]. NIR excites molecular vibrations and thus overtones and combination bands can be observed in a NIR spectrum [15]. The main advantages of NIRS are its comparatively low cost, fast measurements and easy handling [25,26]. Furthermore, it is possible to miniaturize NIR technology and thus reduce costs and weight, as well as improve consumer-friendliness [27,28]. With the miniaturization process beginning already a decade ago, its main challenge was to preserve spectral performance in terms of wavenumber range and resolution [28]. Thanks to technical advances, like the micro electro-mechanical system (MEMS) and the linear variable filter (LVF) technology, the performance of miniaturized devices increased greatly [29]. These technologies are implemented in various miniaturized devices designed for pharmaceutical and chemical industries. But currently, the miniaturization process is even more sophisticated, which makes a pocket-sized NIR spectrometer possible for the first time. Multiple companies (e.g., Tellspec, Consumer Physics and others) have launched NIR spectrometers so small, they fit into the palm of a hand. Additionally, the implementation of those spectrometers into mobile phones is not a fantasy of the future, but already possible now [30]. Small spectrometers like that are usually not targeted to industries, but to consumers, who want to make educated, science-based food choices. But this device could also be used at food competitions, where the judges usually have to rely on the claims of producer and who were, until now, unable to verify those claims. Hence, the operation of this new generation of miniaturized spectrometers is easy and intuitive. The devices can be operated without any knowledge in the field of chemistry or physics.

But the question of the performance of those pocket-sized spectrometers remains. Since they are not targeted at scientists and researchers, there is little knowledge about the performance of these spectrometers. This is why the present study aims at shedding light upon some aspects of the performance of a pocket-sized molecular sensor—The SCiO (Consumer Physics, Tel Aviv, Israel). Only few studies have been published using this device and thus more information is needed [31,32].

SCiO is operated with a smartphone via Bluetooth. SCiO can be controlled using either the SCiO or the SCiO Lab app. The SCiO app contains a set of pre-established calibration models for, for example, calories and water content of fruits and vegetables; and sugar, fat and calories for chocolates. It also contains a pre-established app for water, protein and fat content of dairy products like cheese, yoghurts and puddings. Hence, once a sample is measured all values are directly given to the user, without presenting a spectrum or a model. The main advantage of this app is its easy handling and intuitiveness. A disadvantage of the SCiO app is that only products for which a calibration model already exists can be measured. If the user wants to measure another product, the SCiO Lab app has to be used. There, spectra can be recorded and later be analysed using the cloud-based web application, SCiO Lab.

The current study investigates different cheese samples in terms of their fat and moisture content using a benchtop NIR spectrometer, as well as SCiO. The performance of both devices is compared using statistical parameters, such as the standard error of prediction and the coefficient of determination (R^2^).

## 2. Results

### 2.1. Near-Infrared Spectroscopy

The NIR region is often divided into three sub-regions: Region I ranges from 800–1200 nm (12500–8500 cm^−1^) and is also called the “Herschel” region. It is the only region where electronic transitions can be observed. Furthermore, the Herschel region contains overtones and combination bands. Region II is located from 1200–1800 nm (8500–5500 cm^−1^) and mainly comprises first overtones. Region III ranges from 1800–2500 nm (5500–4000 cm^−1^), where the combination band can be found [23].

**Figure 1:**
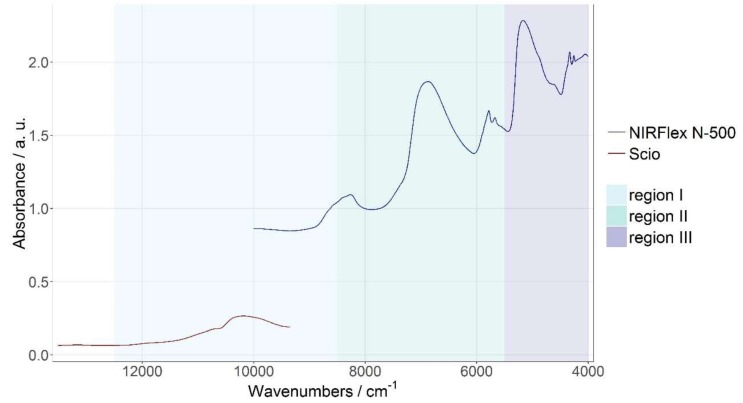
Averaged spectra of whole pieces of cheese of spectra recorded with SCiO (red) and NIRFlex N-500 (blue).

As shown in Figure 1, the spectral range of the benchtop NIRFlex N-500 is 2500–1000 nm (10000–4000 cm^−1^) and thus it mainly includes vibrations in region II and III. The benchtop device also reaches into the Herschel region; however, it does not cover it completely. SCiO on the other hand exclusively records spectra in region I, as its wavelength range reaches from 740–1070 nm (13514–9346 cm^−1^). This also means that SCiO reaches into the visible part of the electromagnetic spectrum. This makes a comparison between the two devices interesting, as the recorded vibrations influence the performance of each spectrometer. The NIR region was chosen as the reference range for various reasons. First of all, molecular vibrations corresponding to water and fat are quite pronounced in this region. Secondly, the different colours of the cheese are irrelevant in this region. Thirdly, using SCiO and the NIRFlex N-500, the same measurement setup and mode could be used. Table 1 lists important peaks in the respective spectra and the corresponding vibrations.

### 2.2. Multivariate Data Analysis

First, all models developed using The Unscrambler X version 10.5 are presented and evaluated. Then the data generated with the SCiO web-application will be examined in detail and, lastly, the performance of the pre-established models from the SCiO app will be discussed.

#### 2.2.1. Regression Models Established with The Unscrambler X Version 10.5

**Figure 2:**
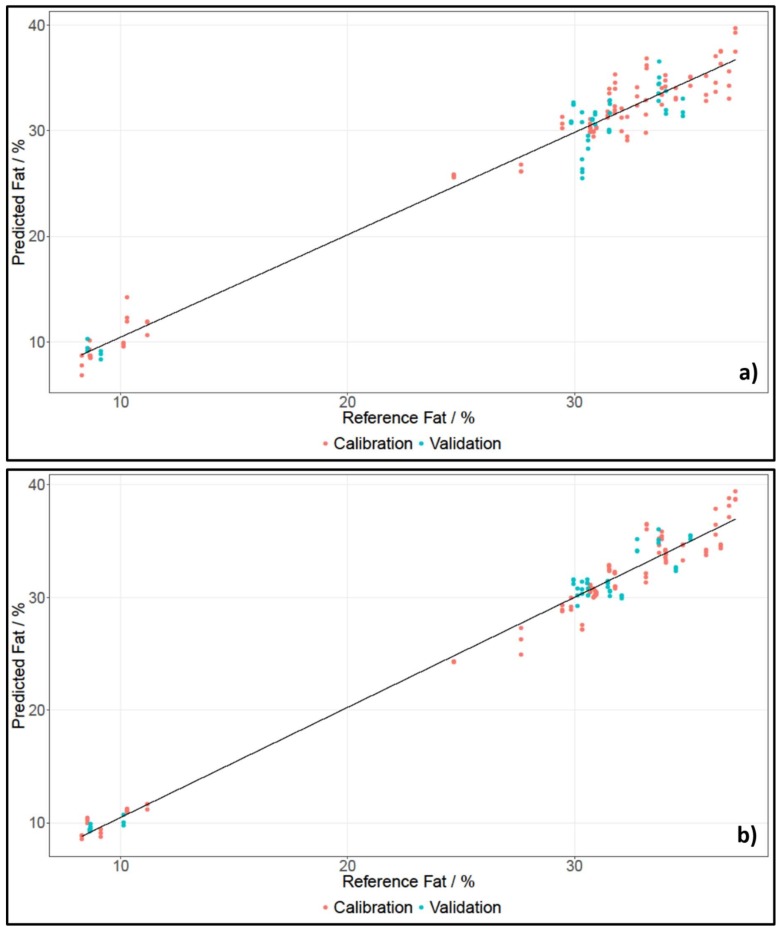
PLS regression of fat content of whole pieces of cheese, established using data of NIRFlex N-500 (**a**) and SCiO (**b**).

For spectral data pre-treatment, the descriptive statistics tool, which is implemented in The Unscrambler X, was consulted and then a fitting pre-treatment was applied (see Section 4.3.1). Table 2 lists the important statistical parameters for the cross- and test set-validated models, calculated using the respective calibration sets for fat content. Figure 2 shows the PLS regressions for whole pieces of cheese for SCiO and the NIRFlex N-500.

For whole pieces of cheese and grated cheese measured with the NIRFlex N-500 and SCiO, two principle components (PCs) were needed to develop calibration models for fat content. Cheese has a high fat content, which is why a low number of principle components for calibration models can be expected. The number of PCs used was determined using the variance plot, which showed how much of the original information is included in the respective PC. Since the same number of PCs were used for calibrations established with SCiO and the benchtop device, as well as for grated and whole pieces of cheese, the results had a high comparability.

The coefficients of determination for all models were between 0.9431 and 0.9940. This indicated a high correlation between the spectra and the reference data. The errors of the cross-validated calibration models, called root mean square errors of cross validation (RMSECV), were between 0.78% and 1.57%, which was acceptable, considering that the average of the reference data was 29.17%. When investigating the test set-validated models, it immediately meets the eye that the Biases were not zero, which is to be expected when using an independent test set. Furthermore, the root mean square error of prediction (RMSEP) values were between 0.77% and 1.90%. All but one RMSEPs were lower than their respective RMSECV. This was most likely due to the Kennard–Stone sample selection, whereby a set of samples was chosen to best represent the multivariate space of the data. Because of that, high value samples, as well as low value samples, were selected in order to best describe the multivariate space. Hence, the error of the calibration set was higher, since it included more extreme values. The independent test set, on the other hand, could then easily be fitted into the well-described multivariate space. Furthermore, all RMSECV values were close to their respective RMSEP values, indicating a robust model.

In general, the best model for the estimation of fat content was the model developed for NIRFlex N-500 data when measuring grated pieces of cheese. The cross-validation and the independent test set-validation both had a coefficient of determination of over 0.99, and the RMSECV and RMSEP were both under 1%. Furthermore, the RMSECV and RMSEP values were close, indicating a robust model. Additionally, the statistical parameters suggested that the analysis of fat content for grated pieces of cheese was slightly better. This was most likely due to the fact that whole pieces of cheese can be spatially inhomogeneous.

**Figure 3:**
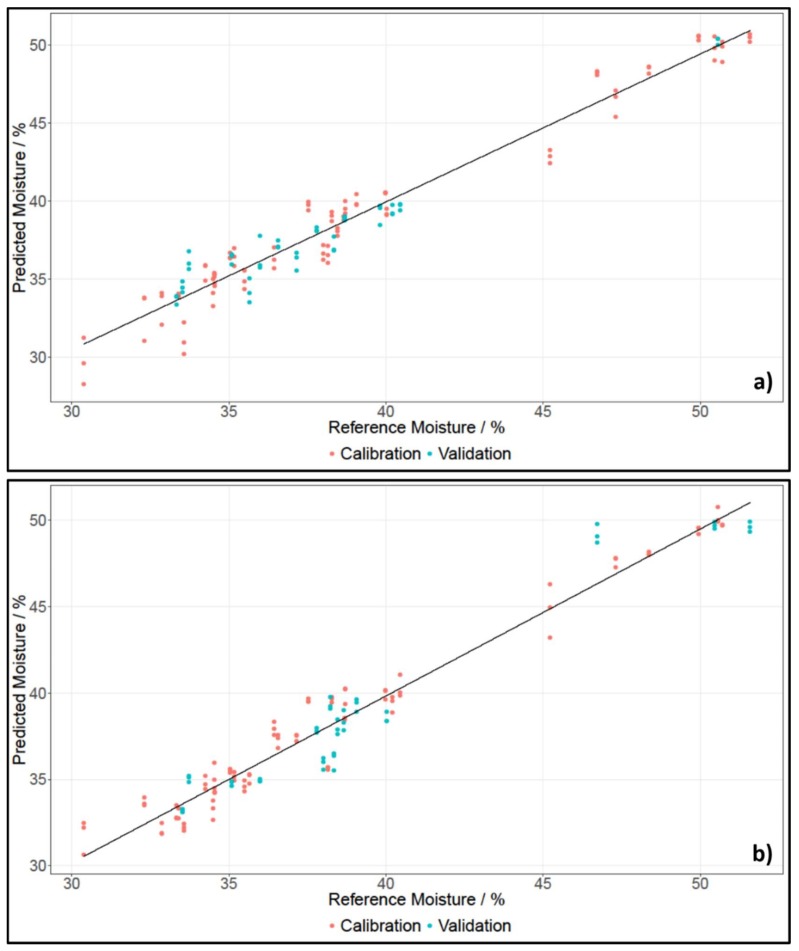
PLS regression of moisture content of whole pieces of cheese, established using data of NIRFlex N-500 (**a**) and SCiO (**b**).

Table 3 lists the results for moisture content for grated cheese and whole pieces measured with the NIRFlex N-500 and SCiO. Figure 3 shows the PLS regressions for whole pieces of cheese for SCiO and the NIRFlex N-500.

For measurements with the NIRFlex N-500, three principle components were needed to develop models for moisture content for whole pieces and grated cheese. For data recorded with SCiO, two principle components were needed to develop models for moisture content. The benchtop device recorded spectra in a broader range than the SCiO, which is why multiple O-H vibrations could be found. However, these vibrations did not only stem from water, but also from other components. The SCiO on the other hand, recorded spectra in a very narrow range, hence only one O-H vibration was visible. Since the spectra of the NIRFlex N-500 were more complex, more principle components had to be used. The number of PCs did not differ greatly though, hence the results could still be compared.

The coefficients of determination for all models were between 0.9327 and 0.9873. This indicated a high correlation between the spectra and the moisture content. The RMSECV values of the cross-validated models were around 1%, with the highest being 1.22%, and the lowest being 0.69%. The RMSEP values for the test set-validated models were also around 1%, with the exception of RMSEP for data recorded with SCiO for grated cheese, which gave a value of 1.71%. Only one RMSEP value was lower than the respective RMSECV value. In general, an error of about 1% for moisture content was quite low, considering that the mean of the reference data was 38.42%.

The difference in prediction accuracy for moisture content of grated cheese and whole pieces of cheese was smaller than for fat content. The prediction of moisture content seemed to work better on grated cheese when working with the NIRFlex N-500; however, when using SCiO, whole pieces showed better prediction results.

#### 2.2.2. Regression Models Established with the SCiO Lab Web Application

SCiO only offers a limited amount of statistical properties. It does not list coefficients of determination for test set-validated models or the Bias of any model. The algorithm for the validation process is proprietary and is; therefore, not accessible for users. The calibration model was validated using leave-one-out cross-validation (LOOCV) and then a test set was also used to validate the model. Table 4 lists the result for the analysis of SCiO data, recorded with the SCiO Lab app, using the SCiO Lab web application.

The coefficients of determination for all cross-validated models were between 0.972 and 0.988, indicating a high correlation between spectra and reference values. Additionally, all root mean square errors (RMSEs) were around 1%, with the lowest being 0.834% and the highest being 0.950%. All but one standard errors of prediction (SEPs) were lower than their respective RMSEs. The lowest SEP was achieved for the prediction of fat content in grated cheese, at 0.779%. The highest SEP was 1.102%. These results were in accordance with the analysis of the same data set using the software The Unscrambler X version 10.5. However, interestingly, the prediction of the fat content with the benchtop device yielded a much higher SEP. Furthermore, different spectral pre-treatments were needed. This is most likely due to the limited options in SCiO Lab. Additionally, SCiO Lab suggested four principle components for all models. Conducting a manual analysis with the Unscrambler X; however, showed that only two PCs explain over 90% of the variance of the original data set. This suggests that the automated algorithm in SCiO Lab takes in too many PCs. This might also be the reason why some of the SEPs were lower for the SCiO Lab web application.

Additionally, it was attempted to imitate the results from the SCiO Lab web application with the Unscrambler X. However, since it was unknown how many smoothing points the SCiO Lab web application applies, results could not be duplicated. The SEP for fat content for whole pieces of cheese was 0.844%, and for water content, 0.825%. This means that for moisture the imitated results, using Unscrambler X, yielded better results, but for fat content the SCiO Lab web application yielded better results. Looking at grated cheese, the SEP for fat content was 0.771% and for moisture content it was 1.400%. This means that the SEP for moisture content was higher than predicted with the SCiO Lab web application, but the fat content was almost the same.

#### 2.2.3. Results from the Pre-Established Model in the SCiO App

For the analysis of the performance of the pre-established model in the SCiO app, all values were registered in Microsoft Excel and then SEPs and Biases were calculated (Table 5).

The Biases for moisture content were quite high, indicating a systematic shift between the data in the pre-established model and the recorded data. The SEPs were all around 1%, but slightly higher than for the self-developed models. This is most likely due to the fact that the model of the SCiO app was developed for “dairy products”, including yoghurt, cheese and puddings.

## 3. Discussion

Reviewing all SEPs and RPDs in Table 2, Table 3, Table 4 and Table 5, the qualities of the calibration model can be estimated. In the Supplementary Material, a table that compresses the most important information can be found. With the help of RPD, the quality of the model can be estimated. RPDs below 2 are not sufficient, whereas an RPD between 2 and 3 is adequate for screening. RPDs between 3 and 5 are satisfactory. If a value over 5 is achieved, the model is estimated to be good. A RPD over 10 indicates an excellent model [34].

Four models reached an RPD value of over 10: The model developed using the SCiO data for data analysis with The Unscrambler X for fat content of grated cheese (10.398); and the models developed using SCiO data for data analysis with the SCiO Lab web application for fat content of whole pieces and grated cheese (11.448 and 10.799, respectively). The highest RPD was yielded for data of fat content collected with the NIRFlex N-500 of grated cheese (14.022). Furthermore, four models had a value between 3 and 5: The models developed using data recorded with SCiO for moisture content of whole pieces of cheese (4.341) and grated cheese (3.208). Additionally, the RPDs for the SCiO data, recorded and evaluated using the SCiO App, was around 4 for moisture content of whole pieces of cheese (4.021) and grated cheese (4.681). All remaining models had an RPD of 5 or higher, indicating that the performances were satisfactory or even good.

Regarding the SEP values, the results for fat content were, overall, better than for moisture. Figure 2 in Section 2.2.1 shows a gap between cheeses with a low and a high fat content. This may have contributed to an enhancement of results because the range of fat content was broadened, which was beneficial, considering how the SEP was calculated. Unfortunately, cheese with a fat content between 12% and 25% were unavailable.

Overall, the measurements of grated cheese seemed to work better, most likely due to the more homogenous nature of grated cheese. However, results did not improve too much, which leads to the question as whether or not the sample preparation is really necessary.

## 4. Materials and Methods

### 4.1. Sample Management and Reference Data

Forty-six cheese samples were analysed for their fat and moisture content using NIRS, as well as traditional wet chemical methods. Twenty samples were classified as hard cheese according to their water content of the fat-free mass, the other 26 samples were classified as semi-hard cheese [35]. Upon receipt, a part of each sample was grated and prepared for reference analysis. The remaining whole piece, as well as some grated cheese, was passed on for spectral analysis. The reference data for fat was collected using the Van Gulik method [36], and the moisture content was calculated from dry mass, which was determined using gravimetry [37]. Great care was taken to always uphold the cold chain and thus keep the samples fresh and unaltered. NIR measurements were conducted for grated cheese, as well as whole pieces using two different NIR spectrometers.

### 4.2. Near-Infrared Spectroscopy

All samples were measured, as grated cheese and whole pieces, with the NIRFlex N-500 (Büchi, Flawil, Switzerland) and SCiO (Consumer Physics, Tel Aviv, Isreal). The latter is a pocket-sized molecular sensor and was launched in 2016. The former is a modular benchtop device.

For measurements of the whole pieces of cheese, the NIRFlex N-500 was operated with the Fibre Optics Solids module. The fibre had an outer diameter of 4 mm and a spectral resolution of 8 cm^−1^. The digital resolution was 4 cm^−1^. All samples were measured at four spots three times, in the range 800–2500 nm (10000–4000 cm^−1^), with 64 scans in diffuse reflection mode. Hence, a total of twelve spectra were received for each sample.

With SCiO, the pieces of cheese were measured at six spots one time in diffuse reflection mode, in the wavelength range 740–1070 nm (13514–9346 cm^−1^), with the SCiO Lab app. The medium resolution of SCiO was 13 cm^−1^, with the lowest resolution (18 cm^−1^) being found at high wavenumbers and the highest resolution (9 cm^−1^) at low wavenumbers.

For measurements of grated cheese, a cylindrical quartz cuvette (h = 25 mm, inner diameter = 31.6 mm) was filled with cheese and used for measurements. Thus, a constant measuring angle and method was assured. Grated cheese was analysed with the NIRFlex N-500 in diffuse reflection mode, with 64 scans in the wavelength range 800–2500 nm (10000–4000 cm^−1^). The spectral resolution was again 8 cm^−1^ and the digital was 4 cm^−1^. The cuvette was constantly rotated during measurement and each sample was analysed six times.

For analysis of grated cheese using SCiO, the quartz cuvette was put onto SCiO and each sample was measured six times using the SCiO Lab app. The cuvette was manually rotated between measurements. Spectra were recorded in the range 740–1070 nm (13514–9346 cm^−1^) and the medium resolution was again 13 cm^−1^. All measurements were taken in diffuse reflection mode.

Additionally, grated cheese and whole pieces were also measured with SCiO, using the pre-establish model from the SCiO app. Samples were again measured six times in the same manner as before.

### 4.3. Multivariate Data Analysis

Spectra recorded with the benchtop NIRFlex N-500 were analysed using the external multivariate data analysis software, The Unscrambler X version 10.5 (Camo Software, Oslo, Norway). Spectra recorded with SCiO, using the SCiO Lab app, were also evaluated using The Unscrambler X version 10.5; however, in addition, they were also analysed using the cloud-based web-application SCiO Lab (Consumer Physics, Tel Aviv, Israel). It offers a limited amount of multivariate data analysis options and was developed for laypeople. SCiO Lab, itself, suggests a certain set of pre-treatments; however, the user can also, him- or her-self, decide which pre-treatments to use when turning on the expert mode. In SCiO Lab it is possible to conduct a standard normal variate (SNV) and to calculate first and second derivatives. Furthermore, the average spectrum can be subtracted and the logarithm can be calculated. It is also possible to select a wavelength range. Additionally, the results given by the SCiO app were transferred to Microsoft Excel (Microsoft Corporation, Redmond, WA, USA) for statistical analysis.

#### 4.3.1. Spectral Pre-Treatments

Spectra recorded with the benchtop device were evaluated using The Unscrambler X version 10.5. For whole pieces of cheese, the twelve spectra of one sample were first averaged by a factor of four, in order to obtain three representative spectra per sample. Next, descriptive statistics, which is an implemented tool in the software, was applied to identify necessary spectral pre-treatments. Standard normal variate (SNV) was applied to reduce multiplicative scatter effects. No other pre-treatments were necessary as they did not improve the model much. Regression models for fat and moisture content were then established. The regression model for fat content considered only the regions in the spectrum where C-H vibrations occur. The two regions considered reached from 6040 to 5440 cm^−1^ (1656–1838 nm) and from 8548 to 8076 cm^−1^ (1170–1238 nm). For moisture content, no wavenumber range was selected, as this did not improve results.

Evaluating spectra of grated cheese recorded with the benchtop NIRFlex N-500, the same route of identifying necessary spectral pre-treatments was taken. First, the six spectra of one sample were averaged by a factor of two, in order to again receive three spectra per sample. Next, descriptive statistics was applied and SNV was again used to remove scatter effects. The same wavenumber region as before was used to establish a regression model for fat content. For moisture content, the whole spectral range was used.

Spectra of whole pieces of cheese and grated cheese recorded with SCiO were first analysed with The Unscrambler X version 10.5. All spectra were first averaged by a factor of two, in order to obtain three spectra per sample. Next, descriptive statistics was applied. Like before, SNV was identified as necessary spectral pre-treatment to remove scatter noise. For fat and moisture content, the whole spectral range (740–1070 nm, 13514–9346 cm^−1^) was used.

When using SCiO Lab for multivariate data analysis, different combinations of the provided spectral pre-treatment options were tried. For moisture and fat content for whole pieces and grated cheese, the same spectral pre-treatments were used, namely 1st derivative followed by SNV.

#### 4.3.2. Regression Models

Using the software, The Unscrambler X version 10.5, for model development, the data sets for whole pieces of cheese and grated cheese, recorded with the NIRFlex N-500 and SCiO, were split into a calibration and a test set using Kennard–Stone sample selection [38], respectively. All calibration sets comprised two thirds of the data (31 samples, 93 spectra), and the test sets consisted of the remaining third (15 samples, 45 spectra). As SCiO Lab does not offer sophisticated data selection algorithms, the same samples as given in The Unscrambler X version 10.5 were selected in the cloud-based web application.

In The Unscrambler X version 10.5, partial least squares regression (PLS-R) with cross-validation was applied to the respective calibration sets to establish calibration models. The models were then validated using test set-validation with the pre-established test sets.

In the SCiO Lab web application, the calibration set was also used to build a PLS-R. Therefore, the data set was manually split into a calibration and validation set. Samples selected corresponded to the sample set created using the Kennard–Stone algorithm in The Unscrambler X version 10.5. Afterwards the developed model was validated using the test set.

For the evaluation of the established PLS-R models, different statistical quality parameters were consulted. R^2^ is a measure of the linearity, RMSECV and RMSEP are indicators of the accuracy of the established model and the Bias can point to methodical errors. The RMSECV is similar to a standard deviation, showing how great the differences between expected and actual values are. The RMSEP denotes the difference between the actual reference value and the predicted value by the established calibration model. Additionally, the RPDs [34] can be used to evaluate the applicability of established models. All mentioned parameters are listed in the result section for all developed PLS-R models.

## 5. Conclusions

This study examines the performance of a new hand-held NIR spectrometer, called SCiO, in comparison to a benchtop device. Forty-six different cheese samples—Grated cheese and whole pieces—were investigated in terms of their moisture and fat content. Additionally, different data analysis routes were taken in order to investigate if a deeper knowledge of chemometrics is necessary for the operation of SCiO, or if the limited tools implemented in the SCiO app and the SCiO Lab web application are sufficient to yield acceptable results.

In general, the analysis of cheese worked better when investigating grated cheese, instead of whole pieces. Furthermore, all calibration models showed high correlation between spectra and reference data. All RPDs indicated that the developed models for whole pieces of cheese were satisfactory to excellent.

This study shows that although overall results do improve by applying more sophisticated multivariate data analysis, the difference is only marginal. This implies that, in the near future, companies could easily use small and cheap NIR devices, with pre-established apps, for quality analysis. This is especially important for small businesses, as often appear in the cheese industry, because they are often unable to afford large instruments or an expert in data analysis.

## Figures and Tables

**Table 1:** Important peaks and their respective vibrations in the spectra recorded with the NIRFlex N-500 and SCiO [5,33].

Device	Vibration	Wavenumber/cm^−1^	Wavelength/nm
NIRFlex N-500	C-H str. 2nd overtones	8888–8068	1125–1240
	O-H str. 1st overtones N-H str. 1st overtones	7264–6068	1377–1648
	C-H str. 1st overtones	5856–5604	1708–1784
	Combination of O-H str. and O-H def., C=O str. 2nd overtones	5404–4784	1850–2090
SCiO	C-H str. 3rd overtones	10,834–10,660	923–938
	N-H str. 2nd overtones and O-H str. 2nd overtones	10,616–9506	942–1052

**Table 2:** Parameters of the established PLS-R models for fat content. CV denotes cross-validated models, whereas TV refers to test set-validated regressions.

Spectrometer	State of the Cheese	R^2^ (CV)	RMSECV/%	PC (CV)	R^2^ (TV)	RMSEP/%	Bias (TV)	PC (TV)	RPD
NIRFlex N-500	Whole pieces	0.9726	1.5711	2	0.9431	1.8964	−0.3369	2	5.109
	Grated cheese	0.9930	0.7845	2	0.9913	0.7676	0.3719	2	14.022
SCiO	Whole pieces	0.9801	1.2466	2	0.9838	1.1874	0.1634	2	7.754
	Grated cheese	0.9838	1.0527	2	0.9940	0.8194	0.1776	2	10.398

R^2^—Coefficient of determination; RMSECV—Root mean square error of cross validation; PC—Principle component; RMSEP—Root mean square error of prediction; RPD—Ratio of performance to deviation.

**Table 3:** Statistical parameterd of the established PLS-R models for moisture content. CV denotes cross-validated models, whereas TV refers to test set validated regressions.

Spectrometer	State of the Cheese	R^2^ (CV)	RMSECV/%	PC (CV)	R^2^ (TV)	RMSEP/%	Bias (TV)	PC (TV)	RPD
NIRFlex N-500	Whole pieces	0.9598	1.2239	3	0.9376	1.0960	0.0408	3	5.597
	Grated cheese	0.9873	0.6868	3	0.9561	0.9337	−0.1843	3	6.697
SCiO	Whole pieces	0.9659	1.0407	2	0.9394	1.1357	−0.3763	2	4.341
	Grated cheese	0.9637	1.0400	2	0.9327	1.7147	0.1297	2	3.208

R^2^—Coefficient of determination; RMSECV—Root mean square error of cross validation; PC—Principle component; RMSEP—Root mean square error of prediction; RPD—Ratio of performance to deviation.

**Table 4:** Statistical parameters of the established PLS-R models for moisture and fat content. CV denotes cross-validated models.

Content	State of the Cheese	PC (CV)	R^2^ (CV)	RMSE/%	SEP/%	RPD
Moisture	Whole pieces	4	0.972	0.949	1.050	5.453
	Grated cheese	4	0.977	0.834	1.102	5.034
Fat	Whole pieces	4	0.988	0.950	0.785	11.448
	Grated cheese	4	0.982	1.118	0.779	10.779

PC—Principle components; R^2^—Coefficient of determination; RMSE—Root mean square error; SEP—Standard error of prediction; RPD—Ratio of performance to deviation.

**Table 5:** SEPs and Biases for the pre-established "dairy products" model in the SCiO app.

Content	State of the Cheese	SEP/%	Bias/%	RPD
Moisture	Whole pieces	1.349	2.266	4.021
	Grated cheese	1.159	2.443	4.681
Fat	Whole pieces	1.064	−0.826	7.832
	Grated cheese	1.218	−0.789	6.844

SEP—Standard error of prediction; RPD—Ratio of performance to deviation.

## Supplementary Materials

The supplementary materials are available online.
