# Supplementary material for: Investigations into the Performance of a Novel Pocket-Sized Near-Infrared Spectrometer for Cheese Analysis
Source: Molecules. 2019 Jan 24;24(3):428. doi: 10.3390/molecules24030428 (PMC6385083; doi:10.3390/molecules24030428)
Supplement: Supplementary file 1 [file molecules-24-00428-s001.pdf]

Supplementary material 1. SEPs and RPDs for all routes of analysis.

Spectrometer	Data analysis software	Content	State of cheese	SEP / %	RPD
NIRFlex N-500	The Unscrambler X	Fat	Whole pieces	1.887	5.109
			Grated cheese	0.679	14.022
		Moisture	Whole pieces	1.108	5.597
			Grated cheese	0.926	6.697
SCiO	The Unscrambler X	Fat	Whole pieces	1.159	7.754
			Grated cheese	0.809	10.398
		Moisture	Whole pieces	1.319	4.341
			Grated cheese	1.729	3.208
	SCiO Lab web-application	Fat	Whole pieces	0.785	11.448
			Grated cheese	0.779	10.799
		Moisture	Whole pieces	1.050	5.453
			Grated cheese	1.102	5.034
	SCiO App	Fat	Whole pieces	1.064	7.832
			Grated cheese	1.218	6.844
		Moisture	Whole pieces	1.349	4.021
			Grated cheese	1.159	4.681

SEP – Standard Error of Prediction; RPD – Ratio of Performance to Deviation.
